# Comparison of invasive and noninvasive blood pressure measurements in critically ill patients receiving norepinephrine

**DOI:** 10.1186/s12871-025-03502-3

**Published:** 2025-12-16

**Authors:** Mahmut Yilmaz, Mete Erdemir, Gurhan Taskin, Levent Yamanel

**Affiliations:** Department of Intensive Care, Gulhane Training and Research Hospital, Ankara, Türkiye

**Keywords:** Hemodynamic monitoring, Blood pressure monitoring, Invasive arterial blood pressure, Norepinephrine, Septic shock

## Abstract

**Background:**

Blood pressure monitoring is crucial in critically ill patients, with invasive arterial blood pressure (IABP) recognized as the gold standard. Noninvasive blood pressure (NIBP) is widely used but may differ from IABP, particularly in patients receiving norepinephrine. This study evaluated the agreement between IABP and NIBP and the impact of the norepinephrine dosage on the accuracy of NIBP in septic shock patients.

**Methods:**

This prospective observational study was conducted in a 36-bed ICU (April 2022–April 2023) and included 84 patients with septic shock receiving norepinephrine. IABP was measured via a radial artery catheter, and NIBP was recorded simultaneously from the contralateral arm. Patients were grouped by norepinephrine dose: ≤ 0.25, 0.25–0.50, and ≥ 0.50 mcg/kg/min. Agreement was assessed via Bland-Altman and error grid analyses, with a focus on the mean blood pressure (MBP) thresholds (≥ 65 mmHg and < 65 mmHg).

**Results:**

Among the 2,104 paired measurements, significant discrepancies were observed between the IABP and NIBP across all dose groups(*p* < 0.001). Discrepancies increased with higher norepinephrine doses, particularly at an MBP ≥ 65 mmHg, with biases of -3.89 mmHg (≤ 0.25 mcg/kg/min) and − 6.81 mmHg (≥ 0.50 mcg/kg/min). Error grid analysis revealed that 47.2% of MBP ≥ 65 mmHg measurements fell into clinically significant risk zones (B-E) at high doses. In patients aged > 65 years, significant differences were observed in all pressures (*p* < 0.001), whereas younger patients showed discrepancies only in the mean and diastolic pressures.

**Conclusions:**

This study demonstrated significant discrepancies between IABP and NIBP in critically ill patients receiving norepinephrine, particularly at higher doses and in patients with MBP ≥ 65 mmHg. These findings highlight the limitations of NIBP monitoring in this population and support the importance of IABP monitoring in selected high-risk patients, while suggesting that NIBP monitoring may remain acceptable in more hemodynamically stable individuals. Applying these considerations in clinical practice may help optimize blood pressure monitoring strategies in the ICU.

**Supplementary Information:**

The online version contains supplementary material available at 10.1186/s12871-025-03502-3.

## Introduction

Blood pressure monitoring is a fundamental component of patient management, providing clinicians with a vital tool for assessing hemodynamic status. Current guidelines for hemodynamic management in circulatory shock recommend maintaining a mean blood pressure (MBP) ≥ 65 mmHg to ensure adequate tissue perfusion [1]. Invasive arterial blood pressure (IABP) monitoring is widely regarded as the gold standard for assessing hemodynamic parameters in critically ill patients in the intensive care unit (ICU) [2]. IABP provides real-time, continuous monitoring and offers the additional advantage of facilitating frequent arterial blood sampling for gas analysis. However, it carries risks such as bleeding, infection, and vascular injury. When IABP monitoring is not feasible, noninvasive blood pressure (NIBP) measurements are used as an alternative, providing intermittent and easily obtainable readings. This method is commonly used in critically ill patients because of its simplicity and accessibility [3]. Therefore, understanding the differences between IABP and NIBP measurements is particularly important in these cases.

Several prospective studies suggest that NIBP readings generally agree with IABP measurements, potentially allowing for their interchangeability [4]. According to the Association for the Advancement of Medical Instrumentation, an acceptable level of agreement between IABP and NIBP in adults is defined as a mean difference of ≤ 5 mmHg and a standard deviation (SD) of ≤ 8 mmHg [4].

Current guidelines recommend norepinephrine as the first-line vasopressor for the treatment of septic shock [5].

However, these effects may also influence the accuracy of noninvasive blood pressure measurements by altering peripheral vascular resistance and arterial stiffness. Although IABP remains the gold standard for hemodynamic monitoring, the clinical feasibility of NIBP in patients receiving vasopressors warrants further investigation, particularly regarding dose-dependent effects. This study hypothesizes that the agreement between IABP and NIBP measurements decreases as the norepinephrine dose increases, potentially leading to clinically significant discrepancies in critically ill patients.

## Methods

**Fig. 1:**
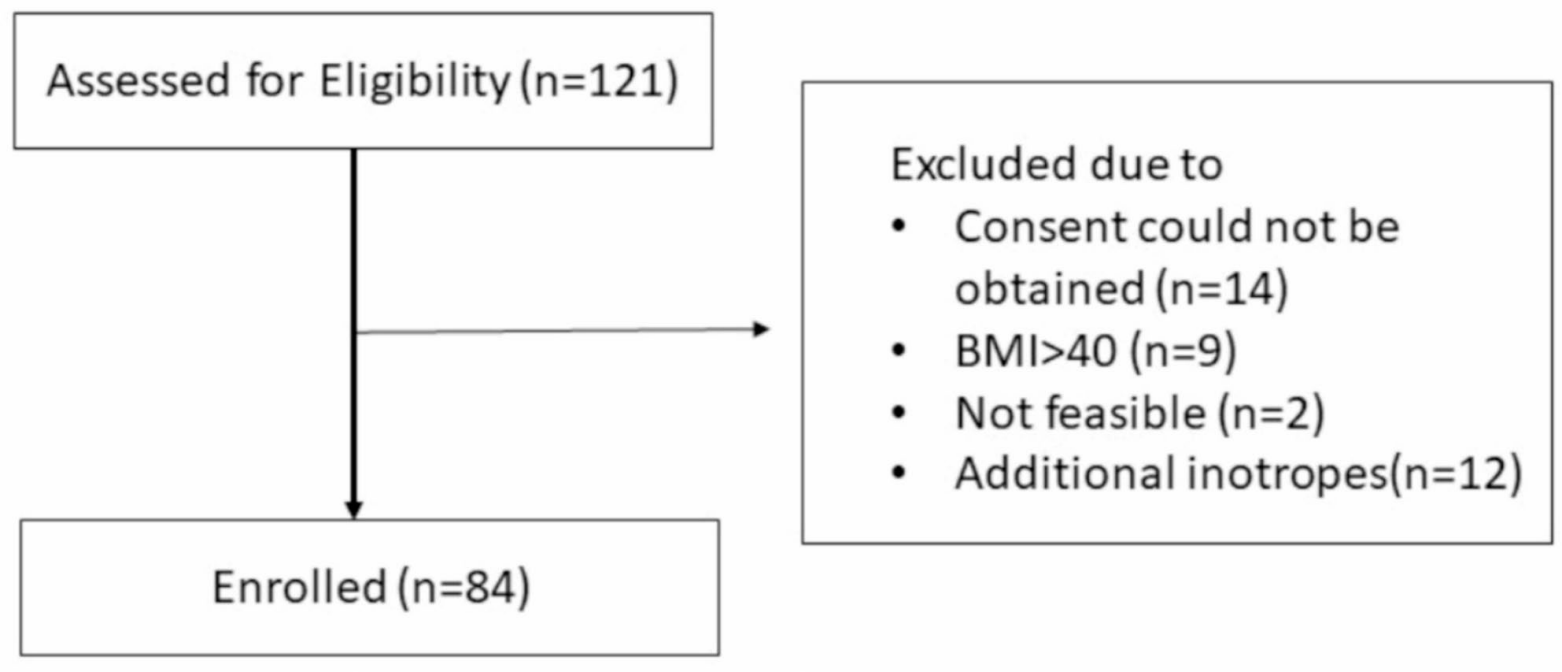
Study flow chart illustrating patient inclusion and data collection. (BMI: body mass index)

This prospective observational study was conducted in a 36-bed, tertiary-level ICU at a teaching hospital between April 2022 and April 2023. Ethical approval for the study was obtained from the local ethics committee. A total of 84 adult patients with septic shock receiving norepinephrine were enrolled in the study (Fig. 1), in accordance with the 2021 Surviving Sepsis Campaign guidelines for sepsis diagnosis [5]. The exclusion criteria were as follows: patients with a body mass index (BMI) greater than 40 kg/m², patients in whom NIBP and/or IABP measurements from the upper extremity were not feasible (e.g., due to amputation or burns), and patients who received either no norepinephrine or additional inotropic agents/vasopressors alongside norepinephrine. Furthermore, patients with conditions known to cause significant side-to-side blood pressure discrepancies, such as aortic dissection or major aortic aneurysm, were not present in the final study cohort.

Invasive arterial blood pressure (IABP) was measured hourly via a 20-G radial artery cannula, connected to a PHILIPS^®^ IntelliVue MX550 monitor using a fluid-filled pressure monitoring system. The arterial transducer was leveled at the mid-axillary line (phlebostatic axis) and zeroed to atmospheric pressure at the start of each recording session. Calibration and waveform fidelity were checked at least once per shift, and the system was monitored for damping or signal artifacts during recordings. Noninvasive blood pressure (NIBP) was obtained simultaneously from the contralateral arm at the same time points using a Philips IntelliVue oscillometric cuff system. NIBP measurements were performed automatically at hourly intervals, with SBP, MBP, and DBP derived from oscillometric pulse detection according to the monitor’s algorithm. Norepinephrine dosages were categorized into three groups: ≤0.25, 0.25–0.50, and ≥ 0.50 mcg/kg/min, based on current hemodynamic management guidelines to reflect clinically relevant ranges for low, moderate, and high-dose vasopressor therapy [5]. Norepinephrine was administered as norepinephrine tartrate (salt form); doses are expressed as norepinephrine base equivalent (mcg/kg/min). Patient demographics, comorbidities, primary diagnosis, site of IABP monitoring, and APACHE II and SOFA scores (assessed within the first 24 h of ICU admission) were documented. Additionally, data on ICU length of stay and mortality were collected. The primary objective of this study was to evaluate the agreement between IABP and NIBP in critically ill patients receiving norepinephrine. A secondary aim was to examine the impact of age, comorbidities, and other variables on the agreement between these two measurement methods.

### Statistical analysis

The normality of the data distribution was evaluated via the Shapiro-Wilk and Kolmogorov-Smirnov tests. Variables with a nonnormal distribution were expressed as medians with interquartile ranges (IQR), whereas normally distributed variables were reported as the means ± standard deviations (SD). Student’s t-test was used to compare normally distributed data, whereas the Mann-Whitney U test was used for nonnormally distributed data. The Wilcoxon signed-rank test was used to evaluate differences between two dependent groups with nonnormally distributed data.

Correlations between noninvasive and invasive blood pressure measurements were illustrated via scatter plots. The Bland-Altman method was used to assess the agreement between IABP and NIBP by plotting the means of the two measurements against their differences. The 95% limits of agreement (LOA) were calculated as the mean difference ± 1.96 × standard deviation of the differences [6]. Additionally, linear regression analysis was conducted to examine the relationship between the differences in IABP and NIBP measurements.

To evaluate the clinical relevance of the observed differences between the two methods, error grid analysis was performed [7]. This analysis classifies each pair of arterial pressure measurements into distinct risk zones (A to E). Zone A indicates no clinical risk, where the test and reference methods do not alter management decisions. Zone B represents low risk, where discrepancies between the test and reference methods may result in benign or no treatment. Zone C corresponds to moderate risk, where differences could lead to unnecessary treatment with moderate but non-life-threatening outcomes. Zone D signifies significant risk, where discrepancies may result in unnecessary treatment with severe, although nonlife-threatening, consequences. Finally, Zone E represents a dangerous risk, where inappropriate treatment due to discrepancies could lead to life-threatening outcomes for the patient. It should be noted that error grid zones are based on modeled clinical risk rather than fixed absolute blood pressure differences; therefore, in some cases, discrepancies greater than 20 mmHg may still appear within a low-risk (green) zone.

## Results

A total of 2,104 paired measurements of IABP and NIBP were obtained from 84 patients receiving norepinephrine. The study population had a mean age of 70.7 years, a mean BMI of 26.3 kg/m², and 75% were intubated. The most common comorbidity was malignancy. The detailed clinical characteristics of the study cohort are summarized in Table 1.

**Table 1:** Clinical characteristics of the study population

Variables	Total (*n* = 84)
Age (years)	70.71 ± 14.96
Male	54 (64.2)
BMI (kg/m2)	26.28 ± 6.97
APACHE II score	30.87 ± 9.52
Comorbidities	
Malignancy	46(54.8)
Congestive heart failure	66 (78.6)
Neurological Disorders	12 (14.3)
Renal disease DM	17(20.2) 50 (59.5)
Invasive ventilation Noninvasive ventilation ISBP (mmHg) NISBP (mmHg) IMBP (mmHg) NIMBP (mmHg) IDBP (mmHg) NIDBP (mmHg)	63 (75) 21 (25) 112.2 + 19.88 114.49 ± 19.98 77.99 ± 12.43 82.89 ± 13.92 60.89 ± 13.05 67.1 ± 14.29

A total of 2,104 paired invasive (IABP) and noninvasive (NIBP) blood pressure measurements were obtained from 84 patients, with an average of 25 measurements per patient

Data are given as the *n* (%) or mean ± SD

*SD *standard deviation* BMI* body mass index, *COPD* chronic obstructive pulmonary disease, *DM* diabetes mellitus, *ISBP* invasive systolic blood pressure, *NISBP* noninvasive systolic blood pressure, *IMBP* invasive mean blood pressure, *NIMBP* noninvasive mean blood pressure, *IDBP* invasive diastolic blood pressure, *NIDBP* noninvasive diastolic blood pressure

Comparison of invasive and non-invasive blood pressure values between patients with and without heart failure revealed that all parameters were significantly lower in the heart failure group (Invasive systolic, diastolic, and mean: *p* < 0.001; noninvasive systolic: *p* = 0.002; noninvasive diastolic and mean: *p* < 0.001).

Significant differences were observed between IABP and NIBP measurements for systolic blood pressure (SBP), mean blood pressure (MBP), and diastolic blood pressure (DBP): SBP, 112.2 ± 19.8 vs. 114.0 ± 19.9 mmHg, *p* < 0.001; MBP, 77.9 ± 12.4 vs. 82.9 ± 13.9 mmHg, *p* < 0.001; DBP, 60.8 ± 13.0 vs. 67.1 ± 14.3 mmHg, *p* < 0.001. Correlation analysis between invasive and non-invasive blood pressure measurements showed a weak-to-moderate association. Spearman correlation coefficients were *r* = 0.538 for SBP, *r* = 0.487 for DBP, and *r* = 0.557 for MBP (all *p* < 0.001, *N* = 2104) (Supplementary Table 1).

In patients over the age of 65, significant differences were observed in SBP, MBP, and DBP between invasive and noninvasive measurements (*p* < 0.001). However, in patients under the age of 65, no significant difference was found in SBP (*p* = 0.58), although significant differences were observed in MBP and DBP (*p* < 0.001).

Patients were divided into three groups based on the norepinephrine dose they were receiving at the time of measurement. Among the total measurements, 51.8% (*n* = 1,090) were taken during the administration of norepinephrine at ≤ 0.25 mcg/kg/min, 21.7% (*n* = 458) during doses between 0.25 and 0.50 mcg/kg/min, and the remaining measurements during ≥ 0.50 mcg/kg/min.

In patients receiving norepinephrine at doses < 0.25 mcg/kg/min (Group 1), no significant difference in SBP was detected between IABP and NIBP (*p* = 0.108); however, significant differences in MBP and DBP were noted (*p* < 0.001). In Group 2 (0.25–0.50 mcg/kg/min), significant differences were observed in across all the measurements (*p* < 0.001). In Group 3 (≥ 0.50 mcg/kg/min), all the measurements also showed significant differences (*p* < 0.001).

Linear regression analysis was performed to evaluate the correlation between systolic and diastolic pressure biases and their respective invasive measurements. A significant relationship was found between these parameters across all groups (Table 2).

**Table 2:** Results obtained from the correlation analysis performed between the groups

Variables		Group 1	Group 2	Group 3
Systolic Blood Pressure	*r*	0.486	0.569	0.66
	r^2^	0.236	0.323	0.435
	p	< 0.001^*^	< 0.001^*^	< 0.001^*^
Diastolic Blood Pressure	*r*	0.497	0.536	0.411
	r^2^	0.247	0.287	0.168
	p	< 0.001^*^	< 0.001^*^	< 0.001^*^
Mean Blood Pressure	*r*	0.591	0.594	0.504
	r^2^ p	0.349 < 0.001^*^	0.352 < 0.001^*^	0.254 < 0.001^*^

* : statistically significant (*p* < 0.05)

**Fig. 2:**
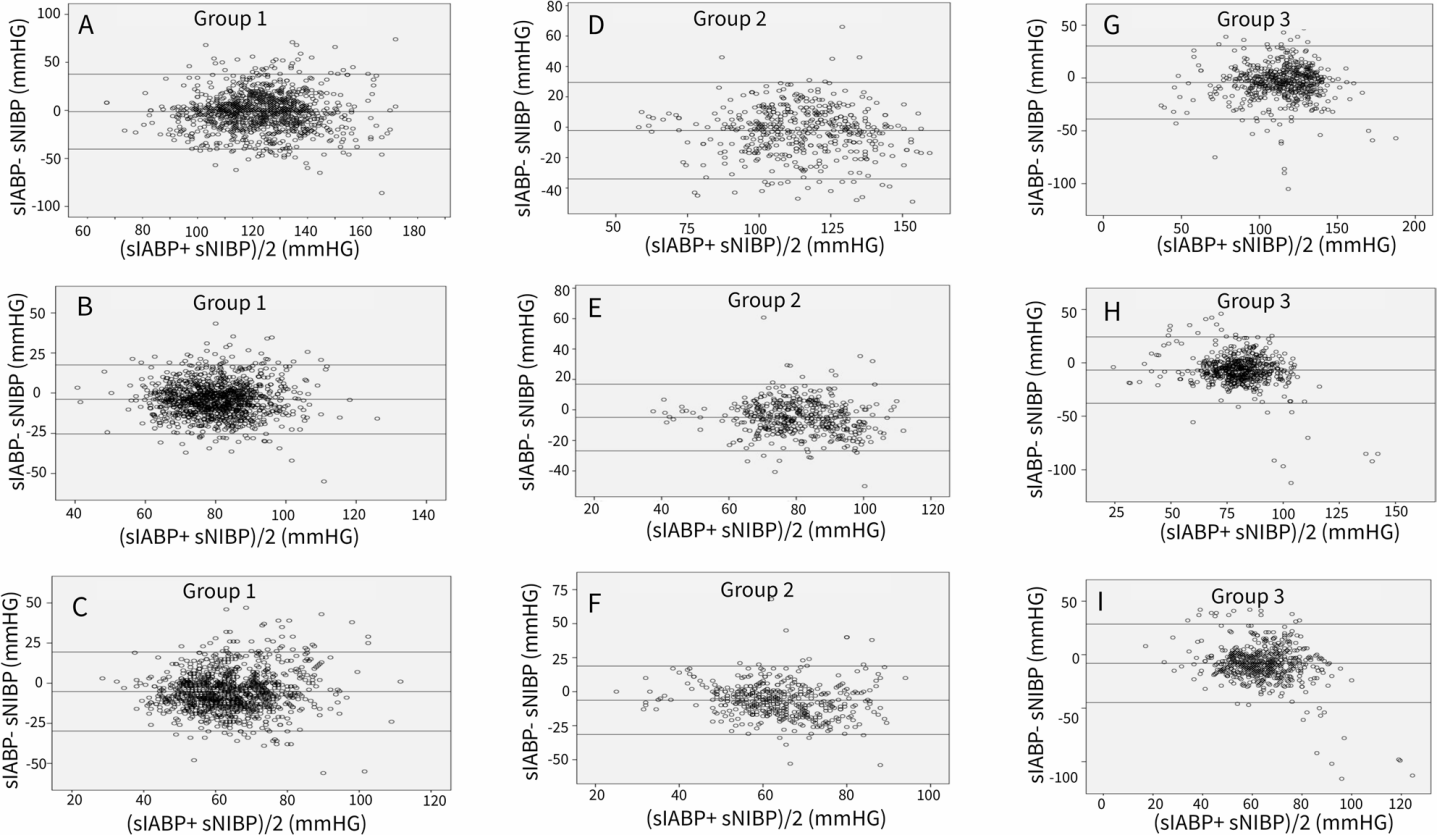
Bland-Altman plots analyzing the agreement between invasive (IABP) and noninvasive (NIBP) blood pressure measurements across different norepinephrine dose groups. Plots **A**–**C** represent Group 1 (< 0.25 mcg/kg/min norepinephrine), plots D–F represent Group 2 (0.25–0.50 mcg/kg/min), and plots **G**–**I** represent Group 3 (≥ 0.50 mcg/kg/min). For each group, systolic (SBP: A, D, G), mean (MBP: B, E, H), and diastolic (DBP: C, F, I) blood pressures are compared. The y-axis shows the difference between IABP and NIBP, and the x-axis shows the average of the two methods. The central solid line represents the mean bias, indicating the average difference between methods, and the dashed lines represent the 95% limits of agreement (± 1.96 standard deviations), showing the range of variability between measurements. (IABP: invasive arterial blood pressure; NIBP: noninvasive blood pressure)

Bland–Altman analysis for SBP, MBP, and DBP showed small mean biases between invasive and non-invasive measurements, with wider 95% limits of agreement at higher norepinephrine doses (Group 1: SBP − 1.3 ± 19.9 mmHg, MBP − 3.89 ± 10.92 mmHg, DBP − 5.19 ± 12.56 mmHg; Group 2: SBP − 2.24 ± 16.2 mmHg, MBP − 4.97 ± 11.13 mmHg, DBP − 6.33 ± 12.83 mmHg; Group 3: SBP − 4.24 ± 17.62 mmHg, MBP − 6.81 ± 15.88 mmHg, DBP − 8.09 ± 18.7 mmHg) (Fig. 2; Supplementary Table 2). To assess the clinical relevance of these mean differences beyond their statistical significance, we calculated Standardized Mean Differences (SMDs; Cohen’s d) for the mean blood pressure. The SMD increased from 0.36 in Group 1 (≤ 0.25 mcg/kg/min) to 0.45 in Group 2 (0.25–0.50 mcg/kg/min), indicating a shift from a small-to-medium to a medium effect size. In Group 3 (≥ 0.50 mcg/kg/min), the SMD was 0.43, maintaining a medium effect size according to Cohen’s convention (where 0.2 represents a small, 0.5 a medium, and 0.8 a large effect).

Following Bland–Altman analysis, linear regression was performed to assess whether the differences between invasive and non-invasive measurements (bias) were associated with the magnitude of blood pressure (proportional bias). This analysis revealed that significant proportional bias was primarily observed for mean and diastolic blood pressures in the higher norepinephrine dose groups (Groups 2 and 3). The complete results of this proportional bias analysis are provided in Supplementary Table 5.

Linear regression analysis revealed a significant proportional bias for MBP and DBP in higher norepinephrine dose groups (Groups 2 and 3), particularly when the MBP was ≥ 65 mmHg (*p* < 0.01 for all), indicating that the difference between IABP and NIBP widened as the blood pressure increased. The full regression statistics for all subgroups are available in Supplementary Table 3. Box plots illustrating the distribution of invasive systolic blood pressure according to age (< 65 vs. ≥ 65 years) and MBP (< 65 vs. ≥ 65 mmHg) are provided in the supplementary material (Supplementary Figs. 1–6).

Error grid analysis was performed for each group. The proportion of measurements in clinically acceptable risk zones (Zone A) decreased as the norepinephrine dose increased. Notably, the proportion of MBP measurements in Zone A dropped from 65.9% in Group 1 to 52.8% in Group 3, while the combined proportion in potentially risky zones (B-E) rose from 34.1% to 47.2%. The complete breakdown for all risk zones and blood pressure parameters is provided in Supplementary Table 4.

**Fig. 3:**
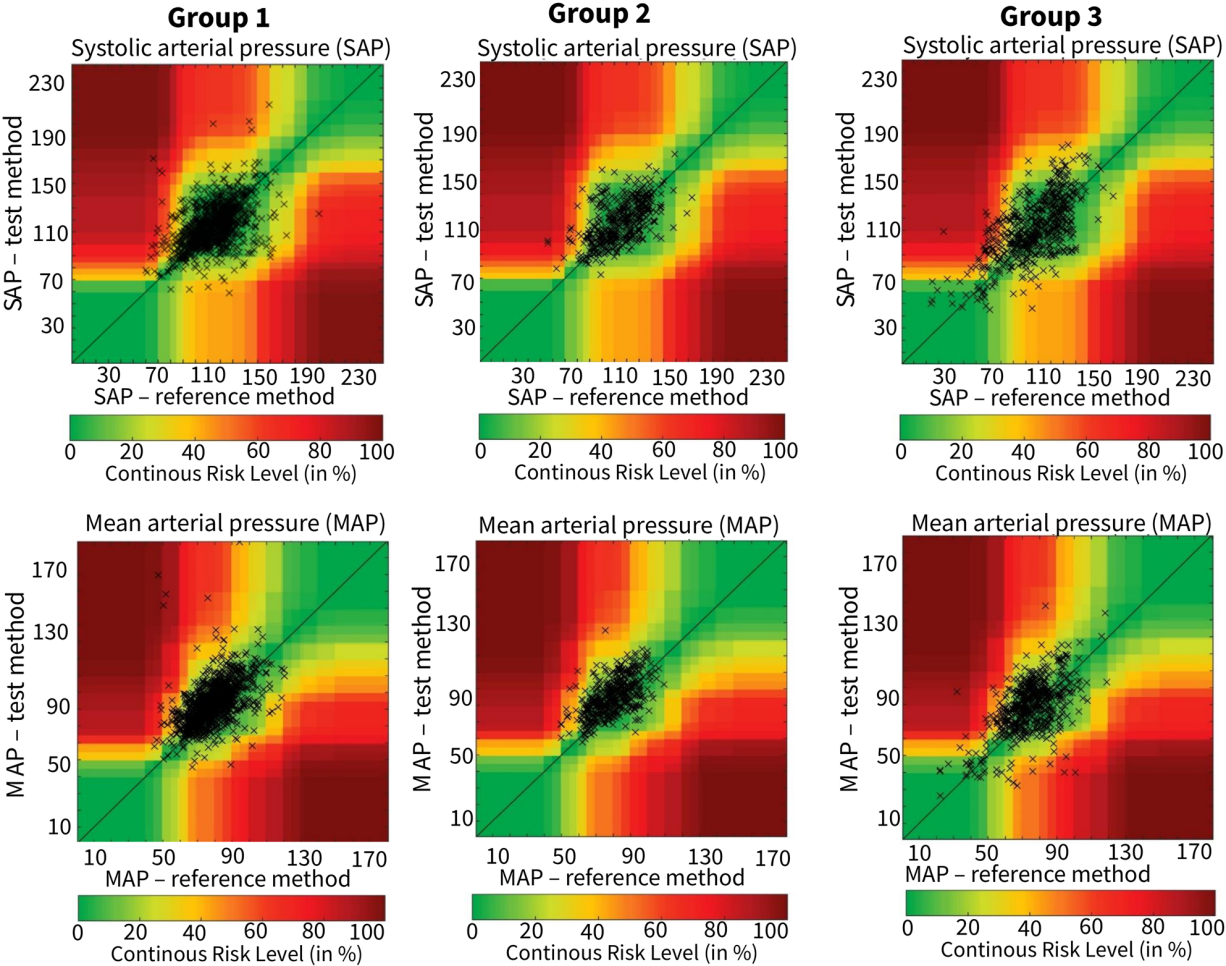
Clinical agreement error-grid analysis for systolic (SBP) and mean arterial pressure (MBP) comparing noninvasive blood pressure (NIBP, test method) to invasive arterial blood pressure (IABP, reference method). Plots A–B: Group 1 (< 0.25 mcg/kg/min), plots C–D: Group 2 (0.25–0.50 mcg/kg/min), plots E–F: Group 3 (≥ 0.50 mcg/kg/min). The zones in the grid represent different levels of clinical risk associated with potential errors when using NIBP instead of IABP (Zone A: minimal risk; Zones B–E: increasing clinical risk). (SBP: systolic blood pressure; MBP: mean blood pressure; IABP: invasive arterial blood pressure; NIBP: noninvasive blood pressure)

Notably, while approximately one in three measurements (34.1%) fell into clinically risky zones (B–E) at low norepinephrine doses (≤ 0.25 mcg/kg/min), this proportion increased to nearly one in two measurements (47.2%) at high-dose norepinephrine (≥ 0.50 mcg/kg/min), underscoring the greater risk of clinically relevant discrepancies with higher vasopressor support (Fig. 3).

**Fig. 4:**
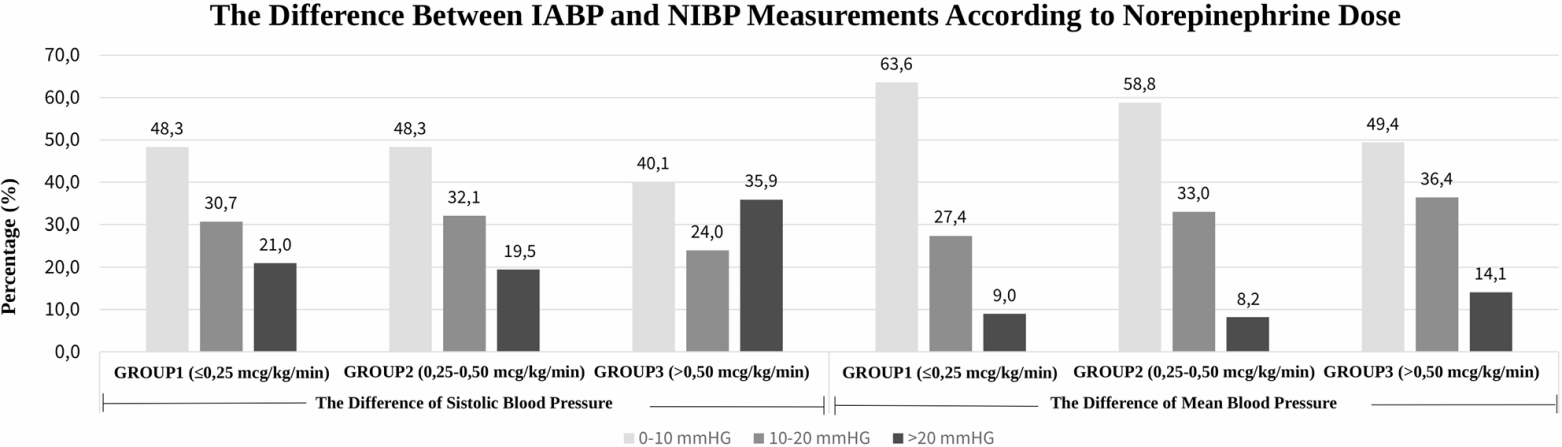
The relationship between norepinephrine infusion dose and the magnitude of the difference between invasive (IABP) and noninvasive (NIBP) blood pressure measurements. The plot demonstrates a trend of increasing discrepancy between IABP and NIBP as norepinephrine dose increases, highlighting the potential impact of higher vasopressor doses on noninvasive measurements. (IABP: invasive arterial blood pressure; NIBP: noninvasive blood pressure)

As the norepinephrine dosage increased, a significant increase in the difference between the IABP and NIBP measurements was observed (*p* < 0.001) (Fig. 4).

## Discussion

In this prospective study, we compared IABP measurements with NIBP measurements in patients receiving norepinephrine treatment. Our findings demonstrate that IABP measurements were significantly lower than NIBP measurements across all parameters. Notably, in patients over the age of 65, significant differences in SBP, MBP, and DBP were observed between the two methods.

In addition to norepinephrine dose and age, underlying cardiovascular comorbidities may influence the discrepancies between invasive and noninvasive blood pressure measurements. Comparison of invasive and non-invasive blood pressure values between patients with and without heart failure revealed that all parameters were significantly lower in the heart failure group. This finding underscores the importance of considering comorbid cardiovascular conditions when interpreting blood pressure measurements in critically ill patients and may have implications for clinical decision-making, particularly in patients receiving vasoactive medications.

In patients with MBP values above 65 mmHg, significant differences were observed between the IABP and NIBP measurements. This MBP threshold is clinically significant, as values above 65 mmHg are generally considered adequate for maintaining organ perfusion [1]. The increasing difference between IABP and NIBP, particularly at high norepinephrine doses, suggests that NIBP becomes less reliable as blood pressure increases.

Patients were categorized into three groups based on their norepinephrine dosage, and a detailed statistical analysis of the differences between the groups was conducted. The threshold values for norepinephrine dosage were determined in accordance with current guidelines [5]. In Group 1 (< 0.25 mcg/kg/min), significant differences were observed in the MBP and DBP but not in the SBP. Following Bland-Altman analysis, linear regression revealed that the observed differences in SBP and MBP in Group 1 could not be attributed to measurement size, a pattern that was similarly observed in Groups 2 (0.25–0.50 mcg/kg/min) and 3 (≥ 0.50 mcg/kg/min). Interestingly, when patients with MBP values of 65 mmHg or higher were analyzed, regression analysis revealed significant differences across all groups. This suggests a nonrandom and systematic relationship between the measurement methods in populations targeting specific hemodynamic goals.

The increasing divergence between IABP and NIBP in patients with MBP values above 65 mmHg suggests that invasive methods are more accurate and reliable. This increasing difference, particularly in patients receiving high doses of norepinephrine, may be attributed to the effects of vasopressor therapy on vascular tone and resistance. High-dose norepinephrine can cause significant peripheral vasoconstriction, potentially leading to inaccurate NIBP readings and clinical errors.

The absence of systematic bias in SBP measurements suggests that both methods may be interchangeable in certain clinical situations. However, caution is warranted when these methods are used in patients receiving norepinephrine doses exceeding 0.25 mcg/kg/min, particularly in intensive care settings where a target MBP of 65 mmHg is often critical. Our findings underscore the need for careful interpretation of blood pressure values in patients receiving high doses of norepinephrine, as the risks associated with IABP monitoring may outweigh the benefits when NIBP can be used safely. Notably, as the norepinephrine dosage increases, the risk of making clinically dangerous decisions rises significantly.

Notably, our findings are consistent with those of Jiang et al., who retrospectively analyzed 96,673 measurements from 6,060 patients and reported similar discrepancies between invasive and noninvasive blood pressure measurements in critically ill patients receiving vasopressors [8]. Other investigations in critically ill adults receiving inotropes documented SBP and DBP differences correlating with the number of inotropes used, while studies in neurocritical care also reported significant discrepancies between invasive and noninvasive measurements under vasoactive therapy [9, 10] Similarly, Meidert et al. highlighted the limitations of noninvasive monitoring in patients with altered vascular tone [11]. Several distinctive aspects differentiate our study from these prior works: its prospective design, detailed stratification across different norepinephrine dose groups (Groups 1–3), separate Bland-Altman analyses for SBP, MBP, and DBP in each group, the use of an error grid to assess the proportion of measurements falling within risk zones, and the additional subgroup analysis of patients below and above 65 years of age.

In a recent meta-analysis by Hasegawa et al., central arterial SBP measurements were on average 8.0 mmHg higher than peripheral (radial or brachial) measurements, and MAP about 3.5 mmHg higher in critically ill patients [12]. These findings highlight the presence of pressure gradients along the arterial tree under conditions of altered vascular tone and wave reflection. In our study, non-invasive SBP was measured at a more proximal site (e.g., brachial cuff), while invasive measurements were obtained from the radial artery. Radial artery measurements are known to underestimate central arterial pressure in patients with septic shock receiving vasopressors, whereas femoral artery monitoring offers a more accurate representation [13]. Therefore, anatomical location, arterial compliance, wave reflection, and vasopressor-induced peripheral vasoconstriction may partially explain the discrepancies observed between invasive and non-invasive measurements. Future studies should consider stratifying by measurement site (e.g., brachial vs. radial vs. femoral) to better clarify these effects.

Bland-Altman analysis provides valuable insights into the accuracy and precision of measurements. However, it is important to note that these analyses may not fully capture the clinical significance of the observed differences. Error grid analysis offers a quantitative assessment of the clinical consequences of differences between the two measurement methods [7, 14]. Although the mean biases observed in Bland–Altman analysis were relatively small (− 1 to − 8 mmHg), the wide limits of agreement (up to ± 40 mmHg) indicate substantial variability at the individual measurement level, highlighting that invasive and non-invasive measurements cannot be considered interchangeable in critically ill patients, particularly at higher norepinephrine doses or in patients with MBP >65 mmHg. While the absolute mean differences (e.g., ~ 5 mmHg for MBP) may appear modest, the SMD analysis reveals a dose-dependent effect that reaches a ‘medium’ magnitude in Groups 2 and 3. This suggests that the observed discrepancy is not negligible when considered relative to the variability in the data. More importantly, the clinical relevance is most accurately captured by our error grid analysis, which demonstrates that a substantial proportion of individual measurements (up to 47.2% in Group 3) fell into clinically significant risk zones, where diagnostic or therapeutic errors could occur. Therefore, we contend that the combination of a non-negligible effect size (SMD) and a high rate of clinically risky individual measurements underscores the clinical importance of our findings, particularly in patients requiring higher vasopressor support.

In Group 1, the majority of measurements fell within risk Zone A, with 77.1% for SBP and 65.9% for MBP, indicating a lower risk of clinically significant differences. In Group 2, these percentages were 79% for SBP and 61.7% for MBP, whereas in Group 3, a decrease in these values was noted, reaching 63.2% for SBP and 52.8% for MBP, indicating an increase in measurements within higher-risk zones (B to E) as the norepinephrine dose increased. These findings suggest that in patients receiving norepinephrine doses below 0.25 mcg/kg/min, one in three measurements (34.1%) fell within risk zones B to E. In contrast, in patients receiving norepinephrine doses of 0.50 mcg/kg/min or higher, one in two measurements (47.2%) fell within these risk zones. Consequently, the likelihood of making clinically risky decisions increases as the norepinephrine dosage increases in patients monitored with NIBP.

Similar to previous studies, these differences are categorized into 10 mmHg increments, with deviations > ± 10 mmHg considered clinically significant and deviations > ± 20 mmHg deemed unacceptable. The literature indicates that differences in blood pressure measurements exceeding 10 mmHg are clinically significant, whereas differences exceeding 20 mmHg, particularly in critically ill patients, are considered unacceptable [15, 16].

Our study demonstrated that IABP measurements were significantly lower than NIBP measurements in critically ill patients receiving norepinephrine. Additionally, the differences in measurements correlated with the dose of norepinephrine administered, and this difference was more pronounced in patients receiving norepinephrine doses exceeding 0.50 mcg/kg/min. Notably, in this group, approximately one-third of the SBP measurements and one-seventh of the MBP measurements showed differences of more than 20 mmHg between the two methods.

The physiological mechanisms underlying the observed differences between IABP and NIBP in patients receiving norepinephrine require further investigation. Compared with noninvasive techniques, the potent peripheral vasoconstrictor effects of norepinephrine, particularly through α1 adrenergic receptors, may contribute to lower invasive measurements [17]. Moreover, as highlighted by Wittrock and others, the effect of arterial stiffness could also be a contributing factor, as norepinephrine infusion may increase central arterial stiffness, thereby affecting blood pressure measurements [18]. Norepinephrine increases arterial stiffness, leading to higher oscillometric SBP readings [19, 20]. Shikau et al. reported a significant association between aging and arterial stiffness [21]. In patients over 65 years of age, arterial stiffness likely explains the differences in SBP, MBP, and DBP measurements. Furthermore, the differences between invasive and noninvasive blood pressure measurements increase significantly with increasing doses of norepinephrine. This trend emphasizes the need for caution when noninvasive measurements are interpreted in critically ill patients receiving high doses of norepinephrine.

In clinical practice, these findings suggest that invasive arterial monitoring should be prioritized in patients receiving high-dose norepinephrine (> 0.25–0.50 mcg/kg/min), in elderly patients with increased arterial stiffness, or when maintaining a target MBP of ≥ 65 mmHg is critical. In contrast, noninvasive monitoring may still be acceptable in patients receiving lower norepinephrine doses, provided that hemodynamic stability is maintained. Incorporating such thresholds into ICU protocols may help reduce the risk of misinterpretation and improve patient safety.

### Study limitations

There are several limitations to this study that should be acknowledged. First, the single-center design and modest sample size limit the generalizability of our findings. Additionally, the arterial site of catheter insertion may affect blood pressure readings; radial artery pressures are known to underestimate central arterial pressures, particularly in patients receiving high-dose norepinephrine, which may partially explain the discrepancies observed between IABP and NIBP measurements. Observer variability in cuff placement and measurement technique may also contribute to differences in NIBP readings. Moreover, repeated measurements from the same patient were analyzed using standard statistical methods without mixed-effects modeling, which could affect the accuracy of the results. The diverse etiologies of sepsis in the cohort may have led to individual hemodynamic variations, potentially affecting the findings. To reduce the risk of catheter-related infections, we opted for radial artery measurements. Finally, we did not have detailed information regarding the presence of atherosclerosis among patients, which could potentially influence blood pressure measurements and the observed discrepancies between IABP and NIBP. Additionally, the primary admission diagnoses of the patients (e.g., septic shock, cardiogenic shock) were not fully detailed, and the potential effects of concomitant medications on blood pressure measurements were not systematically recorded, which may have influenced the observed results. Although no patients with aortic dissection or major aortic aneurysm were included, our study did not systematically screen for subclinical or lesser degrees of aortic pathology that could potentially influence blood pressure discrepancies.

In conclusion, this study revealed significant differences between IABP and NIBP in critically ill patients receiving norepinephrine, particularly at higher doses. Our findings suggest that noninvasive measurements may still be acceptable in patients receiving low-dose norepinephrine (< 0.25 mcg/kg/min), in younger patients (< 65 years), or when the target MBP is ≥ 65 mmHg and hemodynamic stability is maintained. However, IABP should be prioritized in patients receiving higher norepinephrine doses (≥ 0.25–0.50 mcg/kg/min), in elderly patients with increased arterial stiffness, or when strict MBP targets are critical for organ perfusion. Future studies should investigate the physiological mechanisms underlying these discrepancies, including the effects of norepinephrine on vascular tone and arterial stiffness. Additionally, larger, multicenter studies are needed to validate these findings and enhance their generalizability.

## Supplementary Information

Supplementary Material 1.

## Data Availability

The datasets used and/or analyzed during the current study are available from the corresponding author upon reasonable request.

## Abbreviations

IABP: Invasive arterial blood pressure
NIBP: Noninvasive blood pressure
MBP: Mean blood Pressure
SBP: Systolic blood pressure
DBP: Diastolic blood pressure
SD: Standard deviation
BMI: Body mass index
