# Supplementary material for: Comparison of invasive and noninvasive blood pressure measurements in critically ill patients receiving norepinephrine
Source: BMC Anesthesiol. 2025 Dec 16;25:609. doi: 10.1186/s12871-025-03502-3 (PMC12709687; doi:10.1186/s12871-025-03502-3)
Supplement: Supplementary file 1 — Supplementary Material 1. [file 12871_2025_3502_MOESM1_ESM.docx]

**Supplementary Figure 1.** Distribution of invasive systolic blood pressure (ISBP) according to age (<65 vs ≥65 years) and MBP (<65 vs ≥65 mmHg) subgroups.


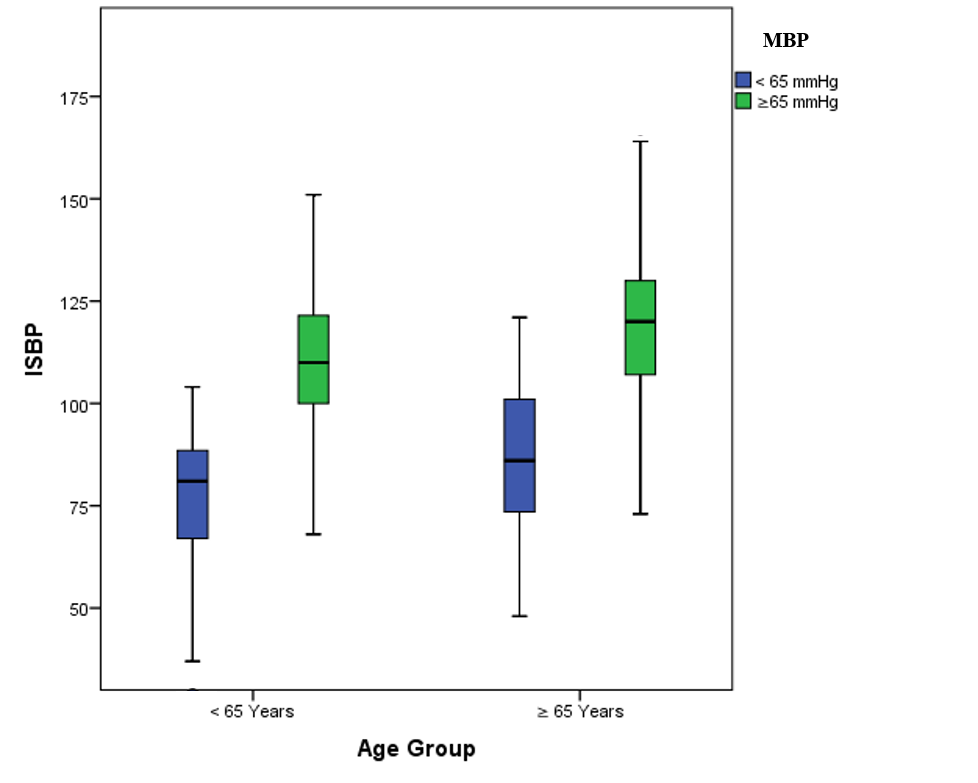


**Supplementary Figure 2.** Distribution of invasive mean blood pressure (IMBP) according to age (<65 vs ≥65 years) and MBP (<65 vs ≥65 mmHg) subgroups.


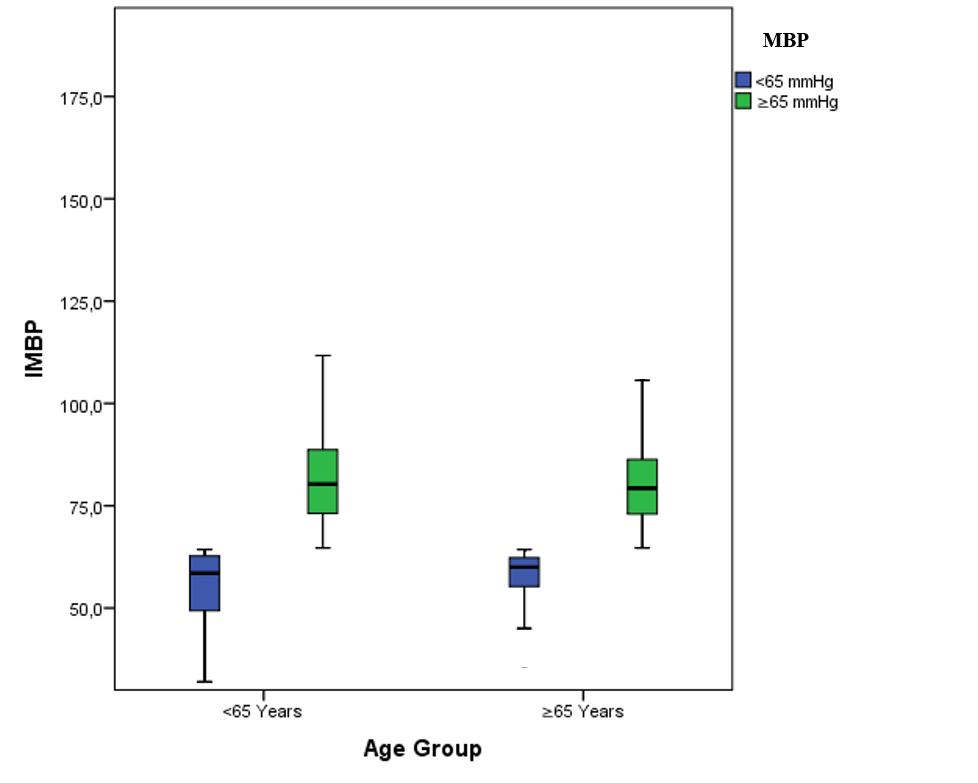


**Supplementary Figure 3.** Distribution of invasive diastolic blood pressure (IDBP) according to age (<65 vs ≥65 years) and MBP (<65 vs ≥65 mmHg) subgroups.


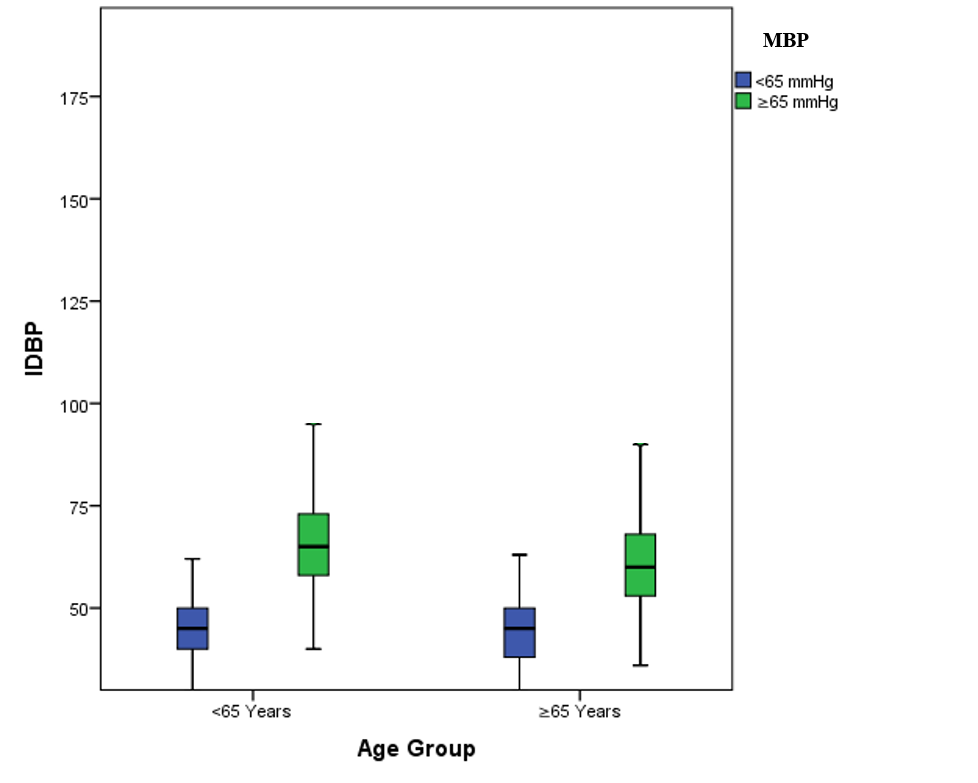


**Supplementary Figure 4.** Distribution of noninvasive systolic blood pressure (NISBP) according to age (<65 vs ≥65 years) and MBP (<65 vs ≥65 mmHg) subgroups.


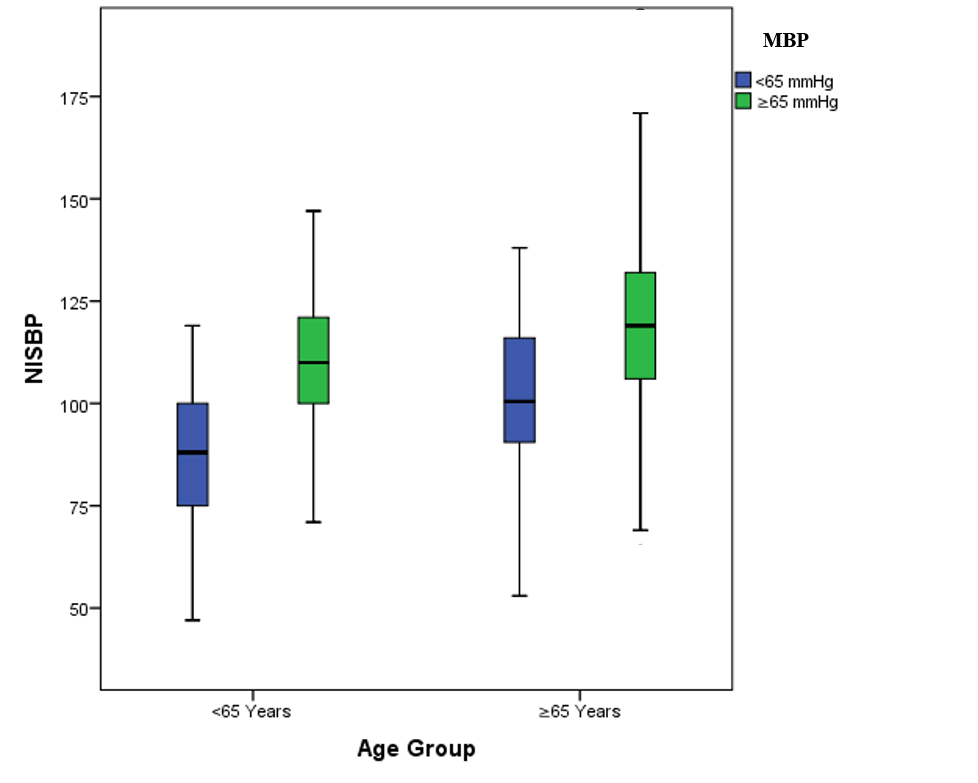


**Supplementary Figure 5.** Distribution of noninvasive mean blood pressure (NIMBP) according to age (<65 vs ≥65 years) and MBP (<65 vs ≥65 mmHg) subgroups.


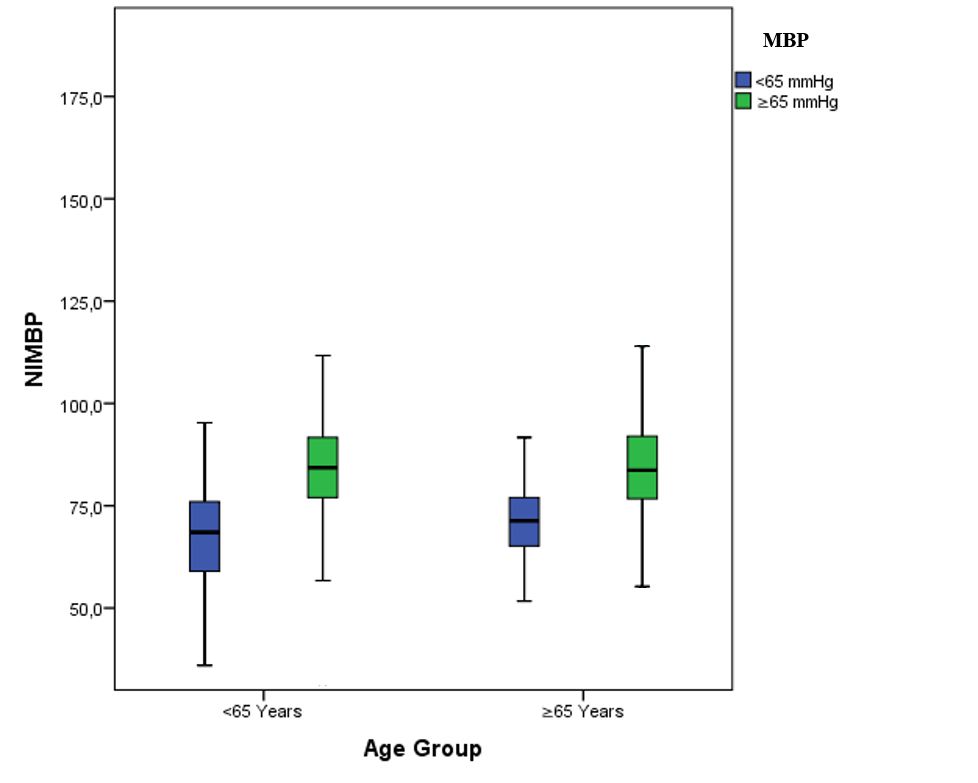


**Supplementary Figure 6.** Distribution of noninvasive diastolic blood pressure (NIDBP) according to age (<65 vs ≥65 years) and MBP (<65 vs ≥65 mmHg) subgroups.


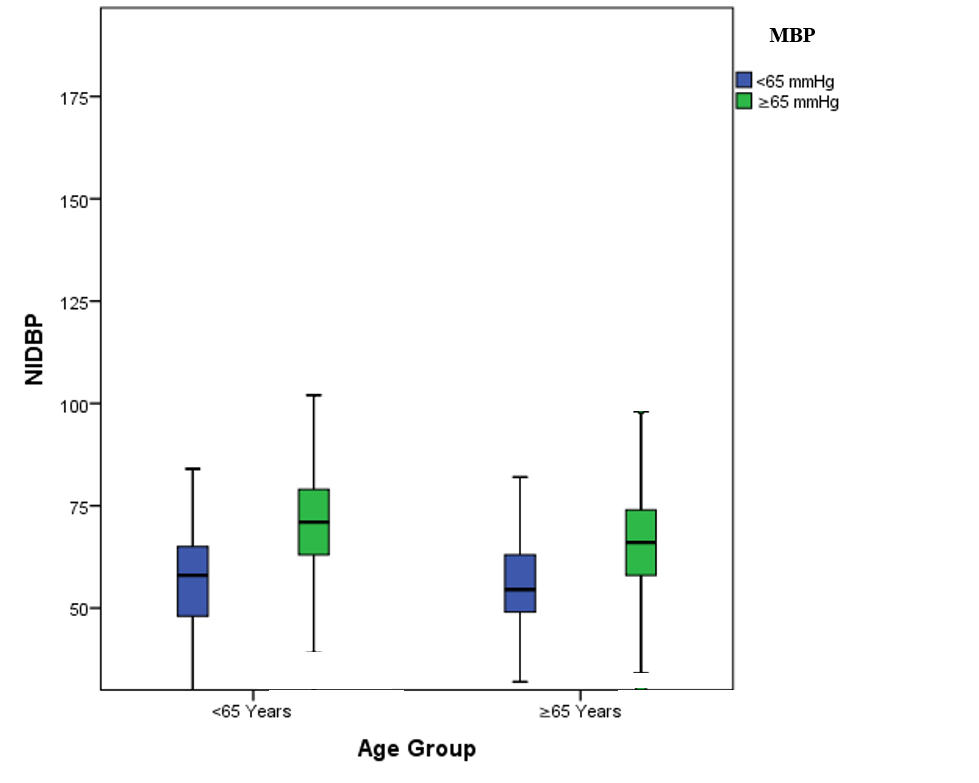


**Table 1.** Spearman correlation between invasive and non-invasive blood pressure measurements (N = 2104)

| **Parameter** | **Spearman r** | **p-value** | **N** |
| --- | --- | --- | --- |
| SBP | 0.538 | <0.001 | 2104 |
| DBP | 0.487 | <0.001 | 2104 |
| MBP | 0.557 | <0.001 | 2104 |

SBP = systolic blood pressure, DBP = diastolic blood pressure, MBP = mean blood pressure

**Table 2.** Bland–Altman analysis of invasive and non-invasive blood pressure measurements by norepinephrine dose groups

| **Group** | **Parameter** | **Bias (mmHg)** | **SD (mmHg)** | **95% Limits of Agreement (mmHg)** |
| --- | --- | --- | --- | --- |
| 1 (<0.25 mcg/kg/min) | SBP | -1.3 | 19.90 | -40.31 to 37.71 |
|  | MBP | -3.89 | 10.92 | -25.31 to 17.52 |
|  | DBP | -5.19 | 12.56 | -29.82 to 19.43 |
| 2 (0.25–0.50 mcg/kg/min) | SBP | -2.24 | 16.20 | -34.00 to 29.11 |
|  | MBP | -4.97 | 11.13 | -26.79 to 16.85 |
|  | DBP | -6.33 | 12.83 | -31.48 to 18.81 |
| 3 (≥0.50 mcg/kg/min) | SBP | -4.24 | 17.62 | -38.78 to 30.28 |
|  | MBP | -6.81 | 15.88 | -37.93 to 24.31 |
|  | DBP | -8.09 | 18.70 | -44.75 to 28.56 |

Measurements were grouped according to norepinephrine dose: Group 1 (<0.25 mcg/kg/min), Group 2 (0.25–0.50 mcg/kg/min), and Group 3 (≥0.50 mcg/kg/min). ias represents the mean difference (IABP - NIBP), with negative values indicating that IABP measurements were lower than NIBP measurements.

SBP = systolic blood pressure, DBP = diastolic blood pressure, MBP = mean blood pressure, SD = standard deviation

**Table 3:** Detailed Linear Regression Analysis of the Relationship Between Mean Blood Pressure and the Difference (Bias) between Invasive (IABP) and Non-Invasive (NIBP) Measurements, Stratified by Norepinephrine Dose and MBP Threshold.

| **Norepinephrine Dose Group** | **MBP Threshold (mmHg)** | **R-value** | **Beta Coefficient (B)** | **P-value** | **95% Confidence Interval for B** |
| --- | --- | --- | --- | --- | --- |
| Group 1 (≤ 0.25 mcg/kg/min) | < 65 | 0.156 | -0.440 | 0.294 | -1.273 to 0.393 |
|  | ≥ 65 | 0.191 | -0.240 | < 0.001 | -0.315 to -0.165 |
| Group 2 (0.25 - 0.50 mcg/kg/min) | < 65 | 0.237 | 0.863 | 0.376 | -1.162 to 2.887 |
|  | ≥ 65 | 0.380 | -0.042 | < 0.01 | -1.44 to -0.61 |
| Group 3 (≥ 0.50 mcg/kg/min) | < 65 | 0.068 | -0.099 | 0.556 | -0.431 to 0.233 |
|  | ≥ 65 | 0.136 | -0.195 | 0.003 | -0.324 to -0.067 |

**Table 4:** Detailed Error Grid Analysis for Invasive versus Non-Invasive Blood Pressure Measurements, Stratified by Norepinephrine Dose.

| **Norepinephrine Dose Group** | **Blood Pressure Parameter** | **Risk Zone A (%)** | **Risk Zone B (%)** | **Risk Zone C (%)** | **Risk Zone D (%)** | **Risk Zone E (%)** |
| --- | --- | --- | --- | --- | --- | --- |
| Group 1 (≤ 0.25 mcg/kg/min) | SBP | 77.1 | 16.6 | 5.4 | 0.45 | 0.27 |
|  | MBP | 65.9 | 31.0 | 2.7 | 0.0 | 2.7 |
| Group 2 (0.25 - 0.50 mcg/kg/min) | SBP | 79.0 | 13.7 | 6.7 | 0.43 | 0.0 |
|  | MBP | 61.7 | 35.1 | 3.0 | 0.0 | 0.0 |
| Group 3 (≥ 0.50 mcg/kg/min) | SBP | 63.2 | 22.1 | 13.3 | 0.89 | 0.35 |
|  | MBP | 52.8 | 41.1 | 5.2 | 0.53 | 0.17 |

This table presents the percentage of paired measurements falling into each risk zone (A-E) of the error grid analysis. Zone A: No risk. Zone B: Low risk. Zone C: Moderate risk. Zone D: Significant risk. Zone E: Dangerous risk.

SBP = systolic blood pressure, MBP = mean blood pressure

**Table 5:** Linear Regression Analysis of Proportional Bias between Invasive and Non-Invasive Blood Pressure Measurements, Stratified by Norepinephrine Dose.

| **Norepinephrine Dose Group** | **Blood Pressure Parameter** | **R-value** | **Beta Coefficient (B)** | **P-value** |
| --- | --- | --- | --- | --- |
| Group 1 (≤ 0.25 mcg/kg/min) | SBP | 0.015 | 0.019 | 0.628 |
|  | MBP | 0.07 | -3.272 | 0.807 |
|  | DBP | 0.091 | 0.103 | 0.003 |
| Group 2 (0.25 - 0.50 mcg/kg/min) | SBP | 0.041 | -0.36 | 0.376 |
|  | MBP | 0.116 | -0.105 | 0.013 |
|  | DBP | 0.133 | -0.151 | 0.004 |
| Group 3 (≥ 0.50 mcg/kg/min) | SBP | 0.013 | -0.012 | 0.753 |
|  | MBP | 0.286 | -0.346 | < 0.001 |
|  | DBP | 0.311 | -0.459 | < 0.001 |

SBP = systolic blood pressure, DBP = diastolic blood pressure, MBP = mean blood pressure
